# Effect of Forward Head Posture on Resting State Brain Function

**DOI:** 10.3390/healthcare12121162

**Published:** 2024-06-07

**Authors:** Ju-Yeon Jung, Yeong-Bae Lee, Chang-Ki Kang

**Affiliations:** 1 Institute for Human Health and Science Convergence, Gachon University, Incheon 21565, Republic of Korea; 9955me@gachon.ac.kr; 2 Neuroscience Research Institute, Gachon University, Incheon 21565, Republic of Korea; 3 Department of Neurology, Gil Medical Center, Gachon University College of Medicine, Incheon 21565, Republic of Korea; 4 Department of Radiological Science, College of Medical Science, Gachon University, Incheon 21936, Republic of Korea

**Keywords:** forward head posture, neutral head posture, cranio-vertebral angle, EEG

## Abstract

Forward head posture (FHP) is a common postural problem experienced by most people. However, its effect on brain activity is still unknown. Accordingly, we aimed to observe changes in brain waves at rest to determine the effect of FHP on the nervous systems. A total of 33 computer users (Male = 17; Female = 16; age = 22.18 ± 1.88) were examined in both FHP and neutral posture. For each session, brain waves were measured for 5 min, and then muscle mechanical properties and cranio-vertebral angle (CVA) were measured. Changes in brain waves between the neutral posture and FHP were prominent in gamma waves. A notable increase was confirmed in the frontal and parietal lobes. That is, eight channels in the frontal lobe and all channels in the parietal lobe showed a significant increase in FHP compared to neutral posture. Additionally, FHP changes were associated with a decrease in CVA (*p* < 0.001), an increase in levator scapulae tone (Right, *p* = 0.014; Left, *p* = 0.001), and an increase in right sternocleidomastoid stiffness (*p* = 0.002), and a decrease in platysma elasticity (Right, *p* = 0.039; Left, *p* = 0.017). The change in CVA was found to have a negative correlation with the gamma activity (P7, *p* = 0.044; P8, *p* = 0.004). Therefore, increased gamma wave activity in FHP appears to be related to CVA decrease due to external force that was applied to the nervous system and cervical spine.

## 1. Introduction

Forward head posture (FHP) is a condition in which the head is translated anteriorly, resulting in sagittal plane misalignment of the cervical spine. It is one of the most common postural deviations and is a representative risk factor for neck pain [1]. In particular, deviation to the center of gravity of the head can increase cantilever loading, which can cause damage to the upper cervical joints and can cause joint instability due to excessive stretching of surrounding muscles and ligaments. Therefore, it is known that FHP can cause various diseases, such as cervical radiculopathy, cervicogenic headaches, and cervicogenic dizziness [2,3]. Recently, it was reported that 78% of the population exhibits deformation of the cervical spine owing to FHP during work due to overuse of smartphones, tablet PCs, and personal computers [4]. In particular, the deformation of FHP that occurs during work is difficult to consciously prevent because it is related to mental concentration to work efficiency [5]. Therefore, problems such as neck pain and disc degeneration associated with continuous stress on the neck and shoulder owing to FHP are continuously increasing [1,6,7,8].

Generally, in a neutral position, the head and shoulders are aligned (earlobe line up with acromion), and the head weight of approximately 10–12 lb is appropriately distributed to the cervical spine [4]. However, when the neck is displaced forward, the weight pressure imposed on the posterior vertebral and muscles increases by more than four times, and the tissues in front of the neck are stretched and strained [9]. Therefore, prolonged FHP and excessive cervical extension increase the load on non-contractile tissues such as vertebrae and ligaments, which are passive subsystems of the cervical spine. In addition, it causes various musculoskeletal pain and dysfunction due to the shortening of extensor muscles and excessive stretching of flexor muscles of the cervical spine [10]. The altered joint position and mechanical load by FHP interfere with the process of sensorimotor control, affecting walking and balance ability. In addition, the slouched posture affects motor responses to cognitive tasks [11,12] and the reduction of respiratory function [13]. These adverse influences caused by mechanical and neural alterations were found to be related not only to motor function but also to cognitive and psychological function [14,15,16,17]. In previous studies, subjects with slouched postures tended to be lethargic and had increased stress and depression [15,16,17]. 

As such, various problems caused by FHP have been studied, and even cognitive and psychological changes have been reported, but to date, not much research has been performed on the effect of FHP on resting brain activity. According to recent research results, it has been reported that changes in brain activity and cognitive function appeared by autonomic nervous system control in relation to body position changes [18,19], but the effects related to head position are not well known. The deformation of cervical alignment can affect brain activity because cranial nerves passed through the cervical spine, such as the vagus nerve, can affect the autonomic nervous system. In particular, among brain waves, gamma’s activity increases due to negative stimulus input such as pain, and as a well-known biomarker of mental stress and depression, it can be expected to be related to FHP [20]. Therefore, in this study, we aimed to investigate the effect of FHP on brain function by comparing and analyzing the brain wave activity that changes in FHP compared to normal posture. In addition, compared to the normal posture, we measure the mechanical changes in muscles that occur in FHP and analyze the relationship between them and brain activity to identify musculoskeletal factors that affect brain function owing to FHP.

## 2. Materials and Methods

### 2.1. Participants 

The sample size of this study was calculated using G*Power version 3.1.9.4. The effect size was 0.601 based on the results of an internal pilot study (n = 10) on changes in the beta power spectrum in resting-state between normal and FHP (mean of the difference = 1.236; standard deviation of the difference = 2.057). According to previous studies, beta activity showed significant differences among postures [19,21]. Based on these findings, we calculated a sample size of 32 for α error probability of 0.05. Considering a potential dropout rate of 10%, 3 more participants were recruited. Thirty-five heavy computer users participated in this study after providing written informed consent. 

As the inclusion criteria, subjects with functional FHP who used visual display terminals for more than 6 h on average per day and whose normal computer use posture was cranio-vertebral angle (CVA) < 50° were recruited. As the exclusion criteria, subjects with a history of musculoskeletal, neurological, or psychiatric disorders and those who experienced any discomfort that might affect the experiment, such as headaches and pain, were excluded from the study. As a result, two subjects without functional FHP (CVA > 50°) were excluded, and their personal information was immediately destroyed. The subjects’ general characteristics (age, height, weight, gender, and functional CVA) are summarized in Table 1. This study was approved by the institutional review board (IRB No. 1044396-202101-HR-015-01) of Gachon University Bioethics Committee and the World Health Organization International Clinical Trials Registry Platform (Clinical Research Information Service (CRIS) number: KCT0007814). The participants were directly recruited by flyers posted in public places in Incheon, Republic of Korea, from 17 February 2021 to 30 December 2021. 

### 2.2. Experimental Protocol and Intervention

**Figure 1:**
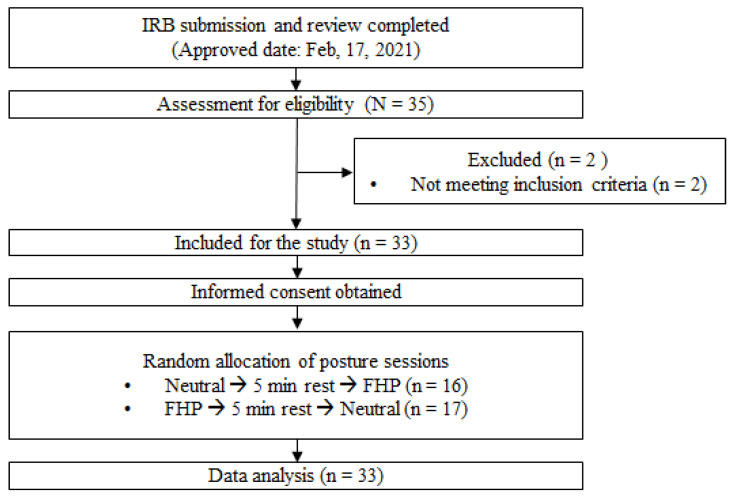
Flow chart of the experimental procedure.

A cross-over study design was performed to confirm changes in electrophysiological functions at rest due to functional FHP. All measurements were performed in neutral posture and functional FHP, and the two postures were applied in random order. Resting EEG measurements were performed for a total of 5 min for each session (neutral posture and functional FHP). After the EEG measurement was completed, muscle mechanical properties (tone, stiffness, and elasticity) and CVA for posture were measured. After all measurements for one session were completed, a 5 min break was taken to wash out the effects of the previous intervention. The above procedure was equally applied in the next session as well (Figure 1).

The following environment was applied by using a setup including an adjustable-height chair with a backrest, ensuring the knees and hips were at a 90° angle with feet placed firmly on the floor [22]. Participants were instructed to place both hands on the keyboard and elbows on the desk comfortably. Additionally, a desktop monitor with adjustable height was used to maintain a vertically downward viewing angle within 10°, positioned approximately 60 cm horizontally from their eyes [12]. 

In functional FHP, participants were instructed to adopt their usual computer posture, which is the typically habituated FHP maintained while using a desktop computer. In order to implement the habituated FHP for computer use, an adaptation time of more than 10 min was provided before the experiment, and measurements were performed after confirming the changed posture. The forward-bent range was set according to individual preferences. 

In the following neutral posture, sufficient training was conducted before the experiment to prevent the subjects from habitual FHP. During this training, they were instructed to sit upright with their backs against the backrest. A cross mark (+) was displayed in the center of the monitor screen, and participants were directed to maintain a normal angle of CVA > 50° while looking at the cross mark and minimizing any forward tilting of their heads as much as possible. Afterward, they underwent familiarization by maintaining this neutral posture for over 10 min before measurements were taken.

### 2.3. Measurements

The participants performed the neutral posture and functional FHP for 5 min each, respectively, and the EEG measurements were applied simultaneously with the intervention. During the EEG measurements, participants were instructed to remain calm and refrain from moving while staring at the center of the monitor, and they were asked to gaze at a cross in the center of the monitor (a white letter in a 60-point font size on a black background) without any distractions. After the intervention, CVA and muscle properties were measured immediately after completion of the intervention while remaining in the intervened posture. A photograph of the participant’s posture was captured to measure CVA. The camera was positioned 1.5 m away from the level of the acromion, and markers were affixed to the anatomical landmarks of the C7 vertebra and the tragus of the ear. The CVA was calculated as the angle formed by the intersection of a horizontal line and the line connecting the C7 spinous process to the tragus of the ear. CVA values were calculated using ImageJ analysis software (Ver. 1.54h) [23]. CVA is a representative method to diagnose the FHP [24]. The muscle properties tone (Hz), stiffness (N/m), and elasticity of the superficial skeletal muscles were measured using a handheld myotonometer (Myoton AS, Tallinn, Estonia) with excellent intra- and inter-tester reliability (ICC = 0.97) [25]. 

Muscle tension (tone), which represents muscle-specific vibration, generally increases as muscle contraction force increases. Stiffness refers to the resistance of muscle tissue to external forces in the initial muscle state. In other words, it means the magnitude of force required to cause displacement of muscle fiber tissue. Elasticity is expressed as a logarithmic decrement and characterizes the dampening of tissue oscillation, meaning that the smaller it is, the higher the elasticity of the muscle. Muscle elasticity refers to the biomechanical properties of a muscle with respect to its ability to return to its initial muscle shape after the reduction or removal of external forces. The muscle properties can be measured at both sides of the suboccipital muscles (SM), levator scapulae (LS), platysma muscle, and sternocleidomastoid (SCM). The exact measurement locations were based on previous studies [7,12,25,26,27,28,29]. The tone and stiffness of all muscles were recorded as average values of three repeated measurements. 

All measurements were conducted in a soundproof room equipped with an electroencephalogram (EEG) at Gachon University. An experienced physiotherapist measured every biosignal. In addition, the order of sessions was blinded to minimize bias of assessments. The sixteen participants performed the neutral posture session followed by the FHP session, while the remaining 17 participants performed the FHP session first, followed by the neutral posture session.

### 2.4. EEG Data Acquisition and Analysis

**Figure 2:**
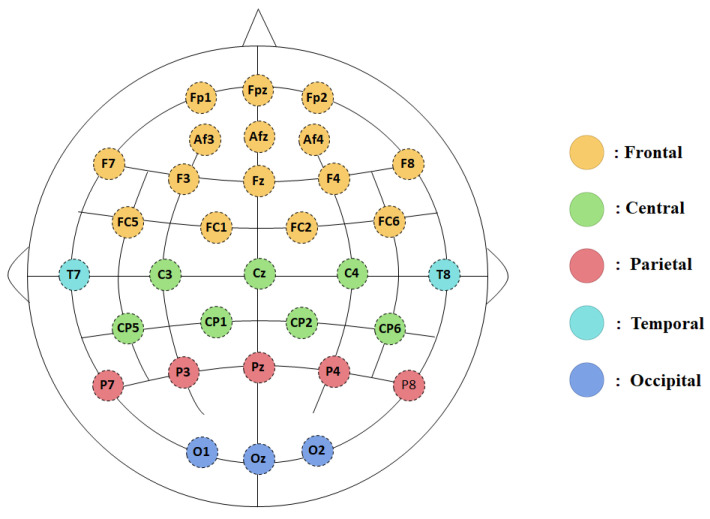
The 32-channel montage representing the grouping of the electrodes into 5 regions.

EEG was measured with 32 active electrodes at locations based on the 10–20 system (QEEG-32Fx, LAXTHA Inc., Daejeon, Republic of Korea), and their positions were as follows: Fp1, FpZ, Fp2, AF3, AF4, AFz, F7, F3, Fz, F4, F8, FC5, FC1, FC2, FC6, T7, C3, Cz, C4, T8, CP1, CP5, CP6, CP2, P7, P3, P4, P8, Pz, O1, Oz, O2. Additionally, both ventral up electrodes and horizontal electrodes were used for the detection of electrooculogram (EOG), and 2 electrodes for electrocardiography (ECG) were used above and below the left subclavian artery. All signals were recorded with TeleScan software (http://laxtha.net/telescan/ (accessed on 23 August 2023)) for 5 min during each session. All impedances of electrodes were kept below 5 kΩ. During data digitization and amplification, an online band pass filter of 0.5–50 Hz was applied. All electrodes were online referenced to A1 and A2 (A1 + A2) during the acquisition of brain waves. Based on a previous study [30], the 32 channels were classified into frontal cortex, central cortex, temporal cortex, parietal cortex, and occipital cortex. In addition, considering the SM noise effect during functional FHP, three channels (O1, O2, and Oz) that were right above the SM were excluded from the analysis (Figure 2).

Independent component analysis (ICA) was performed using MATLAB-based EEGLAB [31] to remove EOG and ECG components. The brain waves used in EEGLAB analysis were pre-defined as follows. First, the data for the first 30 s were removed in order to collect a stable EEG signal. Second, data were re-referenced to the average of all channels without the EOG and ECG electrodes using the reference electrode standardization technique (REST) [32]. Finally, for frequency analysis, fast Fourier transform was used for relative spectral power density (%) of the delta (0.5–4 Hz), theta (4–8 Hz), alpha (8–13 Hz), beta (13–30 Hz), and gamma (30–50 Hz) waves. The relative power spectral density was determined by computing the ratio of delta, theta, alpha, beta, and gamma waves within the frequency range spanning from 0.5 Hz to 50 Hz.

### 2.5. Statistical Analysis

To exclude examiner bias, the experiment and data analysis were performed by different researchers. Jamovi ver.2.2.5 (https://www.jamovi.org/ (accessed on 30 January 2024)) software was used to analyze every biosignal. According to the central limit theorem, as long as the sample is based on 30 or more observations, the sampling distribution of the mean can be safely assumed to be normal [33]. Thus, a parametric statistical analysis method, paired samples t-test, was used to analyze the difference in CVA, muscle properties, and relative power spectrum of EEG. To avoid the multiple comparison problem, the false discovery rate (FDR) was applied to the results of the relative spectral power of the EEG. To examine the relationship between biomechanical changes (i.e., CVA and muscle properties) and brain activity by head position, Pearson’s partial correlation analysis controlling potential confounders (age, sex, height, and weight) was performed. The standard criterion of statistical significance (*p* < 0.05) was applied for all analyses. 

## 3. Results

There were no expected side effects due to the use of safety-proven treatment and measurement equipment for this study. Additionally, because the measurements were conducted for a short period of time (5 min) in the posture that the participants often maintain in their daily lives (FHP and neutral posture), the risk to the subjects was negligible.

### 3.1. Relative Spectral Power Regional Variations

**Figure 3:**
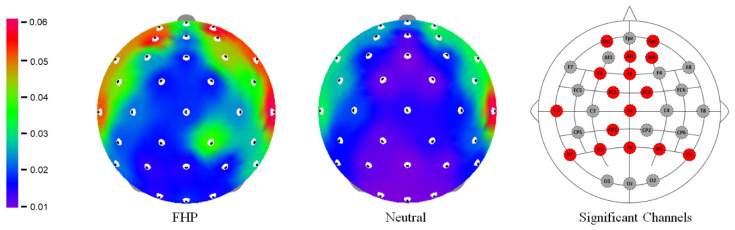
The gamma topographies for the forward head posture (FHP) and neutral posture. The EEG channels with significant differences represent red dots. The color bar indicates the power spectrum value.

Among all brain waves (delta, theta, alpha, beta, and gamma), only the relative spectral power of gamma has passed multiple comparison correction. The results of the relative spectral power of EEG signals showed significant differences, especially in the gamma band, between neutral posture and FHP across all brain regions. A total of 16 channels exhibited significant increases in gamma power during FHP compared to neutral posture. Specifically, eight channels were in the frontal region (FP1, FP2, F3, AF4, AFz, Fz, FC1, and FC2), two channels in the central region (Cz and CP1), one channel in the temporal region (T7), and five channels in the parietal region (P3, P4, Pz, P7, and P8). Among these, the frontal region showed the highest number of significantly increased channels during FHP. In addition, all channels in the parietal region showed significant increases during FHP (Figure 3).

#### 3.1.1. Frontal Region

Gamma oscillations in the frontal region significantly increased during FHP compared to neutral posture. Specifically, gamma power increased by 2.499 in FP1 (T = 2.631, FDR_*p* = 0.037), 3.239 in FP2 (T = 2.465, FDR_*p* = 0.037), 2.086 in F3 (T = 2.876, FDR_*p* = 0.031), 3.119 in AF4 (T = 2.8, FDR_*p* = 0.031), 1.625 in AFz (T = 2.976, FDR_*p* = 0.031), 1.355 in Fz (T = 2.925, FDR_*p* = 0.031), 1.01 in FC1 (T = 2.315, FDR_*p* = 0.049), and 1.713 in FC2 (T = 2.549, FDR_*p* = 0.037) (Table 2).

#### 3.1.2. Parietal Region

All channels in the parietal region (all five channels) exhibited significantly increased gamma oscillations during FHP. Particularly, the highest significant differences were observed in channels P7 and P8. The gamma power increased by 1.809 and 1.716 in P7 and P8 from neutral posture to FHP, respectively (T = 4.42, FDR_*p* = 0.002 and T = 4.294, FDR_*p* = 0.002). Other parietal channels also showed significant increases in gamma power during FHP; P3 (mean difference = 0.927, T = 2.754, FDR_*p* = 0.031), P4 (mean difference = 1.195, T = 2.84, FDR_*p* = 0.031), and Pz (mean difference = 0.783, T = 2.476, FDR_*p* = 0.037) (Table 2).

### 3.2. Biomechanical Changes between Neutral and Forward Head Postures

Table 3 reveals biomechanical alterations associated with FHP, highlighting changes in cervical angle, muscle tone, stiffness, and elasticity, which may have implications for musculoskeletal health.

The mean CVA was significantly lower in the forward head position (39.564 ± 6.390) compared to the neutral position (55.105 ± 4.947) (T = −13.165, *p* < 0.001), indicating a pronounced habituated forward head change occurred during the experiment.

Muscle properties were also significantly affected by postural change. There was a significant increase in both LS tone compared to the FHP with neutral posture. The right LS tone increased by 0.719 Hz (T = 2.598, *p* = 0.014), and the left LS tone by 0.747 Hz (T = 3.498, *p* = 0.001) in FHP compared to neutral posture. The higher frequency observed in the FHP indicates increased muscle tone.

A significantly higher difference in stiffness was observed in the right SCM (T = 3.399, *p* = 0.002) in the FHP (209.485 ± 27.188) compared to the neutral posture (196.848 ± 18.226).

The decrement of both platysma was significantly affected by postural change. The decrement of the right platysma significantly increased by 0.069 (T = 2.158, *p* = 0.039), and the left platysma increased by 0.063 (T = 2.526, *p* = 0.017) in FHP compared to neutral posture. The higher decrement indicates lower elasticity of the muscle.

### 3.3. Correlation Analysis

The power of gamma oscillation with significant differences between neutral posture and FHP had a significant correlation with CVA. Especially, the gamma power occurring in parietal regions (P7, P8) had a negative correlation with CVA (P7, r = −0.266, *p* = 0.044; P8, r = −0.37, *p* = 0.004) (Table 4). On the other hand, the other biomechanical factors (such as tone and stiffness) had no significant correlation with gamma activity.

## 4. Discussion

In this study, we investigated the biomechanical changes in the neck and electrophysiological changes in the brain during FHP. FHP affects not only muscular disorders but also various physiological, psychological, and cognitive problems. Decreased vertebral blood flow, increased stress, and memory decline are associated with FHP. However, the pathophysiological mechanisms for cognitive and psychological problems are not clearly known. In this study, FHP association between mechanical stress of FHP and stressful neurophysiological changes in the brain were observed.

### 4.1. Increased Gamma Activity in FHP

In this study, a significant increase in gamma activity in the overall brain area was confirmed in FHP compared to neutral posture. This suggests that the head position acts as a stressor on the brain and neural tissue during rest. In FHP, the increased forward translation increases the load on the cervical joint and decreases cerebral blood flow due to an interrupted vertebral artery [14]. In addition, the joint stresses and strains transmit abnormal proprioceptive afferent information (deafferentation) to the brain, which may influence the increased gamma activity [34]. Furthermore, this disturbed afferentation process may have an influence on spine-related autonomic dysfunction. The anterior translated spine can increase sympathetic tone and decrease parasympathetic activity by increasing adverse mechanical tension on parasympathetic organs such as the brainstem and cranial nerve 10. In fact, a previous study reported that sympathetic skin response significantly increased in FHP compared to a neutral posture [24]. Since gamma waves are known to be affected by sympathetic modulation and negative stimuli, the abnormal afferent information and altered autonomic nervous system may be a mechanism for increasing gamma activity [18,35].

The increased gamma activity during rest is a sign of abnormal brain activity. In general, default mode network (DMN) and alpha waves play a strong role in normal brain activity at rest. The DMN not only contributes to internal processes such as mind wondering, mental time travel, and perspective shifting but also plays a role in processing long-term information received in daily life [36]. Additionally, alpha waves generally increased during rest are highly related to relaxation, focused attention, and dominant relaxed arousal states [37], and have a significant correlation with the DMN [38]. Therefore, increased gamma activity at rest interferes with normal resting brain activity. The activity of gamma waves during rest is related to the abnormal excitatory system and hyperarousal of the sensory system and can affect the overall level of neural excitation, causing unnecessary arousal and interfering with psychological relaxation [35,39,40,41,42,43]. 

This study also showed a significant negative association between CVA and gamma activity. This suggests that as CVA decreases, distorted sensorimotor information in the cervical spine and longitudinal stress and tension in neural elements increase, which may affect abnormal gamma activity. In addition, gamma activity showed no significant correlation with other muscle mechanical properties except CVA. In this study, reduced elasticity due to stretched platysma and excessive contraction of SCM and LS were confirmed, but no significant correlation with EEG activity was found. This suggests that changes in brain wave activity may be more influenced by changes in joint position than by changes in muscle properties. Therefore, maintaining the normal position of the joint and reducing abnormal neural stress may be more effective in recovering brain function during normal rest.

### 4.2. Muscular Stress in FHP

Changes in muscle properties, such as cervical muscle tension, stiffness, and elasticity, showed similar patterns to previous research results. In general, FHP is accompanied by flexion of the lower-level cervical spine, extension of the upper-level cervical spine, shoulder elevation, and kyphotic thoracic posture [44,45,46], which showed a similar pattern with upper crossed syndrome. In upper crossed syndrome, suboccipitalis, SCM, and LS are tightened, whereas deep neck flexors are stretched [47]. Therefore, excessive tightening and shortening of the SCM and LS reduce the elasticity of the SCM and increase the tone of LS. In addition, stretched cervical flexor muscles lead to a decrease in the elasticity of the platysma. These changes are the same in heavy VDT users with FHP, and a significant increase in bilateral LS tone and stiffness of SCM were reported during computer work [12]. 

However, there were several limitations in this study. First, this study was conducted on healthy adults with functional FHP and only observed the effects in a short-term (5 min) single trial. Thus, the results cannot be representative of chronic subjects with musculoskeletal pain or structural deformities of FHP. Chronic stress, fatigue, and pain caused by structural deformation are negative stimuli and may have a greater adverse effect on changes in brain function at rest, so verification is needed in future research. Additionally, the CVA measured in this study is a highly reliable method of measuring head alignment, through which increased load on the joint and mechanical deformation can be inferred, but changes in actual spine-related dysfunction were not confirmed. Therefore, if a head repositioning accuracy test and an autonomic nervous system test related to spine-related dysfunction in FHP are performed in the future, a more accurate analysis of the effects on brain function will be possible. Finally, the physical activity levels would influence the effects of FHP. For subjects with high physical activity, a neutral posture can be better maintained, and resistance to external force may be high. Therefore, it is necessary to investigate the difference in the impact of FHP between groups with high and low physical activity levels in the future.

## 5. Conclusions

In this study, we observed an increase in gamma activity at rest in FHP, and as a result, it was confirmed that the anterior translated angle of the cervical joint had a significantly negative correlation with the increase in gamma activity at rest rather than muscle stress caused by FHP. This suggests that FHP may interfere with brain function at rest and increase stress-related brain activity. Additionally, it can serve as a basis for clinical intervention and treatments to alleviate neuropsychiatric disorders such as depression, in which gamma wave activity is excessive. Therefore, this study may be helpful in understanding the pathophysiological mechanisms of cognitive and psychological clinical problems caused by FHP by reporting the effects of structural changes associated with FHP on brain activity. In addition, the effects of mental health care can be expected through FHP correction. In fact, it has been reported that depression and sadness can induce changes in posture, and postural assessment and treatment can be effective in diagnosing and treating depression [48]. Therefore, the effects of posture correction treatment (manual therapy, traction, and so on) on the psychological state and brain activity would be effective in patients with depression.

## Figures and Tables

**Table 1:** General characteristics of participants.

	Total (n = 33)	Men (n = 17)	Women (n = 16)
Age (years)	22.18 ± 1.88	22.88 ± 2.26	21.44 ± 0.96
Height (cm)	169.95 ± 8.15	176.26 ± 5.56	162.80 ± 2.93
Weight (kg)	67.14 ± 12.43	74.50 ± 10.60	59.33 ± 9.14
Functional CVA (°)	39.65 ± 6.31	36.94 ± 5.94	42.52 ± 5.49

Abbreviations: CVA, cranio-vertebral angle.

**Table 2:** Comparison of the gamma relative power spectrum between neutral and forward head postures.

Regions	Variables	Forward Head (Mean ± SD)	Neutral (Mean ± SD)	T	FDR_*p*
Frontal	FP1	7.027 ± 7.737	4.528 ± 4.669	2.631	0.037
	FP2	7.836 ± 8.029	4.597 ± 4.255	2.465	0.037
	F3	6.109 ± 6.181	4.023 ± 3.851	2.876	0.031
	AF4	8.443 ± 7.381	5.324 ± 4.777	2.800	0.031
	AFz	4.972 ± 5.136	3.347 ± 3.638	2.976	0.031
	Fz	3.89 ± 3.778	2.535 ± 2.116	2.925	0.031
	FC1	3.581 ± 3.487	2.571 ± 2.026	2.315	0.049
	FC2	4.295 ± 4.314	2.582 ± 2.067	2.549	0.037
Central	CP1	3.082 ± 2.778	2.131 ± 1.713	2.498	0.037
	Cz	2.993 ± 2.582	2.083 ± 1.624	2.471	0.037
Temporal	T7	10.404 ± 8.506	7.158 ± 7.036	2.984	0.031
Parietal	P7	4.746 ± 3.821	2.937 ± 2.299	4.420	0.002
	P8	4.509 ± 3.809	2.793 ± 2.45	4.294	0.002
	P3	3.262 ± 2.417	2.335 ± 1.669	2.754	0.031
	P4	3.569 ± 3.035	2.374 ± 1.759	2.840	0.031
	Pz	2.663 ± 2.204	1.880 ± 1.493	2.476	0.037

Abbreviations: AF, anterior frontal; AFz, midline of anterior frontal; CP, centroparietal; Cz, midline of central; F, frontal; FC, frontocentral; FP, prefrontal; Fz, midline of frontal; P, parietal; Pz, midline of parietal; SD, standard deviation; T, temporal.

**Table 3:** Comparison of the cervical angle and muscle properties between forward head and neutral postures.

Variables	Forward Head (Mean ± SD)	Neutral (Mean ± SD)	T	*p*
CVA (°)	39.564 ± 6.39	55.105 ± 4.947	−13.165	<0.001
Tone_R_LS (Hz)	17.925 ± 1.7	17.206 ± 1.527	2.598	0.014
Tone_L_LS (Hz)	18.25 ± 1.708	17.503 ± 1.922	3.498	0.001
Stiffness_R_SCM (N/m)	209.485 ± 27.188	196.848 ± 18.226	3.399	0.002
Elasticitiy_R_Platysma	1.421 ± 0.243	1.352 ± 0.2	2.158	0.039
Elasticity_L_Platysma	1.398 ± 0.198	1.335 ± 0.142	2.526	0.017

Abbreviations: CVA, cranio-vertebral angle; L, left; LS, levator scapulae; R, right; SCM, sternocleidomastoid; SD, standard deviation.

**Table 4:** Correlation analysis results between gamma power and CVA.

CVA							
Regions	Variables	Pearson’s r	*p*	Regions	Variables	Pearson’s r	*p*
Frontal	FP1	−0.132	0.322	Central	CP1	−0.174	0.193
	FP2	−0.190	0.152		Cz	−0.174	0.192
	F3	−0.122	0.361	Temporal	T7	−0.144	0.281
	AF4	−0.152	0.255	Parietal	P7	−0.266	0.044 *
	AFz	−0.121	0.366		P8	−0.370	0.004 *
	Fz	−0.132	0.324		P3	−0.170	0.201
	FC1	−0.078	0.559		P4	−0.216	0.104
	FC2	−0.212	0.110		Pz	−0.157	0.240

Abbreviations: CVA, cranio-vertebral angle; AF, anterior frontal; AFz, midline of anterior frontal; CP, centroparietal; Cz, midline of central; F, frontal; FC, frontocentral; FP, prefrontal; Fz, midline of frontal; P, parietal; Pz, midline of parietal; T, temporal. * Statistically significant difference: *p* < 0.05.

## Data Availability

The data presented in this study are available on request from the corresponding author. The data are not publicly available due to privacy.
